# Changes in energetic metabolism and lysosomal destruction in the skeletal muscle and cardiac tissues of pigeons (*Columba* livia f. urbana) from urban areas of the northern Pomeranian region (Poland)

**DOI:** 10.1007/s10646-021-02423-4

**Published:** 2021-06-02

**Authors:** Halyna Tkachenko, Natalia Kurhaluk, Tomasz Hetmański, Agnieszka Włodarkiewicz, Vladimir Tomin

**Affiliations:** 1 Department of Biology, Institute of Biology and Earth Sciences, Pomeranian University in Słupsk, Słupsk, Poland; 2 Department of Earth Sciences, Institute of Biology and Earth Sciences, Pomeranian University in Słupsk, Słupsk, Poland; 3 Department of Physics, Institute of Science and Technology, Pomeranian University in Słupsk, Słupsk, Poland

**Keywords:** Energetic metabolism, Oxidative stress, Elements, Pigeons, Motorway, Environmental pollution

## Abstract

The aim of the present study was to evaluate the biochemical responses of the skeletal muscle and cardiac tissues of the urban pigeon as a bioindicator organism tested in diverse environments (Szpęgawa as a rural environment and Słupsk as an urban environment, Pomeranian Voivodeship, northern Poland), resulting in changes in the level of lipid peroxidation at the initial and final stages of this process and the activities of Krebs cycle enzymes (succinate dehydrogenase, pyruvate dehydrogenase, isocitrate dehydrogenase, and alfa-ketoglutarate dehydrogenase). Szpęgawa village was chosen due to the intensive use of the European motorway A1 with significant traffic and pollution levels. The concentration of Pb was higher in the soil and feathers of pigeons nesting in the polluted areas (Szpęgawa). Our studies have shown that the presence of lead in soil and feathers of the pigeons resulted in the activation of lipid peroxidation, destabilization and increased activity of lysosomal membranes, and activation of mitochondrial enzymes of the Krebs cycle with energy deficiency (reduction of ATP levels) in cardiac and skeletal muscle tissues simultaneously.

## Introduction

Involved in the global process of anthropogenic transformation of ecosystems, birds, as their components, inevitably enter the processes of synanthropization and urbanization, acquiring several environmental features. Urbanization is synanthropization in the urban environment, i.e. the process of birds entering from natural landscapes and adapting to the urban environment (Liu et al. 2010). Some studies confirm that birds have an adaptive ability to feed, nest, behave and carry out other living activities in anthropogenic landscapes. To a large extent, this applies to pigeons living next to humans (Dauwe et al. 2003; Grúz et al. 2019).

The adaptation of birds representing different species is expressed in changes in their habitat and nesting habits, breeding biology, nutrition, behavior, territorial changes, tolerance of urban noise, and a shorter scare distance (Lodenius and Solonen 2013). The pigeon (*Columba livia* f. *urbana*) is one of the first birds attracted by human farming. The species belongs to a group comprising synanthropists and urbanists. Part of the pigeon population has lost the ability to exist outside anthropogenic landscapes. Urban environmental issues have increasingly being studied; hence, there are many data on the adaptive mechanisms developed by pigeons as bioindicators in various environments, especially those affected by anthropopressure (Kurhalyuk et al. 2007; Cai and Calisi 2016). Pigeons are adapted to live in close contact with humans; hence, scientific research on ecotoxicology and biomonitoring studies different aspects of the avifauna of urbanized landscapes.

The growth of industrial activity associated with the construction and exploitation of the already existing motorways and the road traffic, in general, has resulted in serious air pollution not only in large cities but also in small villages. In cities and villages, car emission components make a significant contribution to air pollution, which can be identified using effective biosystematics monitoring methods, such as investigations of birds (Ewen et al. 2009). As reported by the authors, its principal source has been attributed to motor vehicles and increasing inner-city congestion, which has to lead to a change and enlargement of transport stop-start zones (Ewen et al. 2009). Vehicle discharges include carbon monoxide, unburned hydrocarbons, nitrogen oxides, volatile organic substances such as benzene and toluene, and heavy metals such as lead (Pb).

Suitable biological indicators for high traffic density areas in different regions of the world are associated with the use of pigeons in biomonitoring studies. Pigeons have been used as indicators of Pb concentrations in the environment. Cai and Calisi (2016) assessed the feral pigeon (*Columba livia*) as a lead bioindicator in New York City and the association with elevated blood lead levels in children. Bala and co-workers (2020) have monitored the concentrations of six toxic metals in industrial areas of India using pigeon excrements as a biomonitor. Among industrial areas and industrial areas vs. reference areas, the trace metal concentrations differ significantly and this suggests a different exposure in these sites to toxic chemicals released from nearby industries, traffic, urban areas, and agricultural land (Bala et al. 2020). Romero and co-workers (2020) have evaluated the exposure to Pb and its relationship with lead-based ammunition in seven species of terrestrial game birds: common woodpigeon (*Columba palumbus*), rock dove (*Columba livia*), stock dove (*Columba oenas*), European turtle-dove (*Streptopelia turtur*), red-legged partridge (*Alectoris rufa*), Barbary partridge (*Alectoris barbara*), and common quail (*Coturnix coturnix*) from rural and urban areas in different parts of Spain. There appears to be a relationship between the hepatic concentrations of Pb in pigeons and their life in urban environments. The rock dove was suggested as a good indicator of contamination in urban environments, while the common woodpigeon could be a good indicator in rural environments (Romero et al. 2020). Soft tissues, bones, and feathers are helpful in permanent monitoring of the homeostasis of chemical elements in birds. Metals can cause oxidative stress by increasing the formation of reactive oxygen species (ROS), which render antioxidants incapable of defense against growing amounts of free radicals (Koivula et al. 2011). Metal toxicity is related to their oxidative state and reactivity with other compounds (Koivula and Eeva 2010; Pizzino et al. 2017).

The data mentioned above data were based on the fact that pigeons, as a synanthropic species, have a fairly small habitat, small body size, high metabolism, and high inhalation rates. This bird species can therefore be used as a biological indicator of environmental pollution (Schilderman et al. 1997). The main routes of exposure shown in some works (Cui et al. 2018) include inhalation of polluted air (Pei et al. 2017) and entry of food and soil particles contaminated with deposited airborne pollutants (Kouddane et al. 2016). Of particular importance are small polished stones in the stomach, called gastroliths. Two important issues in the pigeon ecology and physiology related to the possibility of the use of their feathers as bioindicators should be underlined. Firstly, pigeons spend most of the day feeding (from 25.28% in summer to 38.1% in autumn). The time spent on this type of activity varies greatly between different groups of pigeons: from 17 min to 5 h. Secondly, pigeons do not spend more than one and a half hours a day flying, which is the most energy-intensive activity. Usually, they do not fly more than 800 m away from their nests. Half of the flights are connected with the anxiety factor (Hetmański 2007, 2011).

According to modern concepts, the energy exchange is the leading metabolic link in the body’s vital functions (Dong et al. 2012; Abu Zeid et al. 2019). The mechanism of energy generation is localized in the cell, where the problems of energy synthesis and distribution between energy-dependent processes are solved. In normal physiological conditions, the law of maintaining energy homeostasis, i.e. balancing energy production in energy-synthetic systems and its use in energy consumption reactions, is preserved both at the level of the whole organism and at the level of the cell (Xu et al. 2020). This presupposes the existence of special regulatory systems that control energy consumption and energy-producing processes, the dysregulation of which can cause pathological conditions (Xie et al. 2018). All this makes the problem of regulating energy metabolism and maintaining and preserving energy homeostasis in the cell and at the level of the body exceptional and requires investigation and elucidation of the mechanisms of its disturbance (Wan et al. 2018).

Many researchers report that, in polluted environments, toxic metals and organic pollutants resulting from increased anthropopressure are found to be modulators of the activity of the most important metabolic enzymes (Kamiński et al. 2009; Wołonciej et al. 2016; Koim-Puchowska et al. 2020). ROS generation patterns have a major impact on cellular functions and mainly induce disintegration of cell membrane permeability as well as nuclear and mitochondrial DNA damage (Schilderman et al. 1997). They can also lead to uncontrolled cell division or intensify apoptosis. The redox status, energy changes in enzymes and metabolites involved in the Krebs cycle, and the function of lysosomes in response to environmental stress remain a very important motive for further research into the adaptability of organisms.

Therefore, the main aims of our work were (i) to determine whether the metal content differs between soil and feathers of pigeons living in different contaminated areas; (ii) to compare the activities of alanyl aminopeptidase, leucyl aminopeptidase, β-N-acetylglucosaminidase, and acid phosphatase in various tissues (heart, muscles) of pigeons from different areas of living; (iii) to evaluate the relationship between the activity of Krebs cycle enzymes (succinate dehydrogenase, pyruvate dehydrogenase, isocitrate dehydrogenase, and α-ketoglutarate dehydrogenase) and destabilization of the functioning of lysosomes in cardiac and skeletal muscle tissues of birds nesting in diverse environments; (iv) to estimate the relationship between lysosomal activity and oxidative stress biomarkers estimated by the levels of lipid peroxidation at the first and final stages of the process and the total antioxidant capacity; (v) to assess the relationship of the levels of elements, activity of Krebs cycle enzymes, and energy metabolites (lactate and pyruvate) with oxidative stress biomarkers; (vi) to characterize the trend of main effects (i.e., the environment and type of tissue) in the formation of oxidative stress biomarkers, lysosomal functioning, and element contents in the muscle tissue and the interaction of their effects simultaneously; (vii) to evaluate important determinants of the antioxidant defence, lysosomal stability, and metal levels in these conditions.

## Materials and methods

### Study Area

**Fig. 1:**
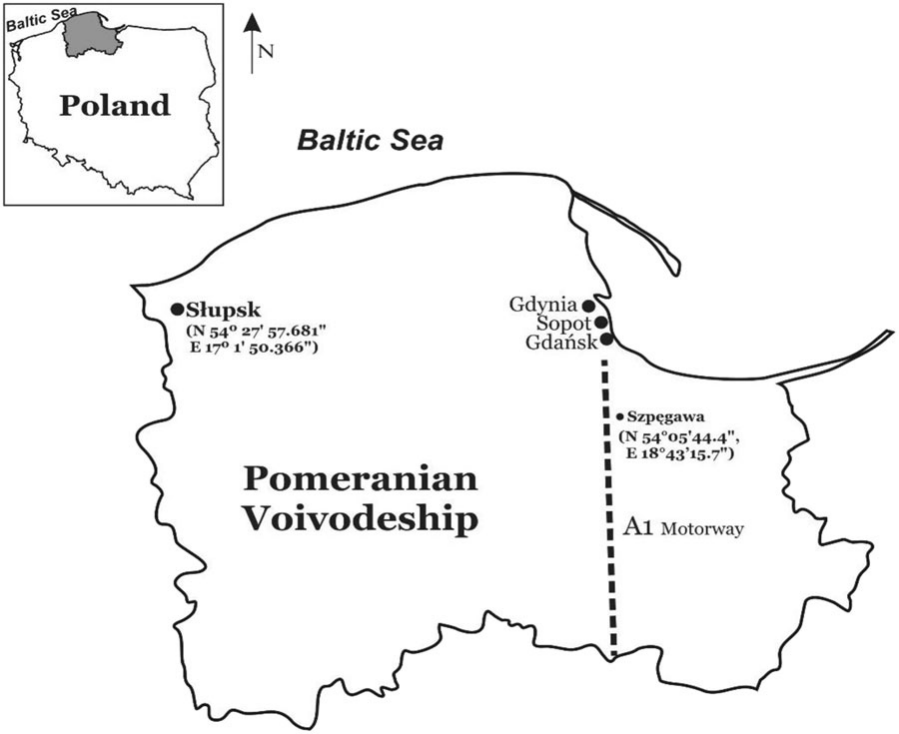
Map of the study area. Szpęgawa (N 54°05′44.4ʺ, E 18°43′15.7ʺ) located near the A1 motorway and Słupsk (N 54° 27′ 57.681ʺ E 17° 1′ 50.366ʺ) in the Pomeranian Voivodeship are marked

The research was conducted in Słupsk and Szpęgawa located in the Pomeranian Voivodeship, northern Poland (Fig. 1). Słupsk (N 54° 27′ 57.681ʺ E 17° 1′ 50.366ʺ) is a city with 90 thousand inhabitants located in the central part of Pomerania. In the central part of the city is the Old Market, a recreational and tourist center. At the same time, the Old Market has been a feeding ground for the largest flock of the urban pigeon (*Columba livia* f. *urbana*) with above 300–400 individuals for many years. The first pigeons appeared in Słupsk in the 1980s. The area where the urban pigeons feed is partly paved; the soil for the urban vegetation has been brought from the neighboring agricultural areas.

Szpęgawa is a village located in the Pomeranian Voivodeship by the Voivodeship road No. 224 in northern Poland. The Stanisławie road junction of the A1 motorway is located near the village. The A1 motorway in Poland is part of the international route E75 in the trans-European transport corridor. It is the only Polish motorway with a southern route. The A1 motorway is characterized by heavy traffic. Currently, it connects the Tricity (Gdańsk, Gdynia, Sopot), i.e., a large metropolitan center, with the Czech border (D1 motorway). One of the problems on such highways is that they are accessed from narrow single-lane roads. As a result, there are huge numbers of slow-moving cars waiting to enter the highway. A busy road leading from the east of the country to the A1 motorway runs through Szpęgawa and Stanisławie. Nearby areas, such as Szpęgawa village in rural areas and the Stanisławie road junction are highly affected by emission of pollutants into the atmosphere, which is also related to the increased volume of freight traffic on international highways as well as the growing popularity of car tourism. In terms of ecological safety, these conditions are regarded as dangerous technogenic systems, with incomplete and uneven combustion of fuel as the main cause of air pollution. It is suggested that only 15% of fuel is used for driving the car, while 85% is emitted into the atmosphere as an aerosol mixture of fuel and combustion products.

Szpęgawa is located about 120 km east of Słupsk. The village includes farms located on agricultural land. For the study of suburban pigeons, a farm located in an area with coordinates N 54°05′44.4ʺ, E 18°43′15.7ʺ was selected. This old farm was established after World War II. To harden the ground on the farm, loose slag (trail), i.e. a waste material from metallurgical production, was used in the 1980s. The farm also is a breeding site of urban pigeons originating from the pigeon population from Słupsk. The colony was established in 2007–2008 to conduct a series of experimental studies, the results of which have been published elsewhere (Hetmański 2011).

### Sampling

In Słupsk, 12 soil samples (each sample was analyzed in three replicates) were taken from the Old Market square, where the largest flock of pigeons is located. In Szpęgawa, 12 soil samples (analysis of each sample was carried out in three replications) were taken from an area between farm buildings where the birds receive food and water. The soil samples were collected from topsoil at the depth of 1–3 cm using a soil sampler. In each of the two sites (Słupsk and Szpęgawa), four sub-sites were established at the perimeter of the area and three soil samples were collected from each of the four sub-sites. Twelve samples, i.e. one from each stated site, were transferred into polyethylene bags and transported to the laboratory. The samples were air-dried for one week, ground to pass through a 200-mesh sieve, and transferred to polyethylene bags until analysis.

Tissue samples were collected from 31 adult birds aged minimum 1 year representing both sexes (17 males and 14 females), with weights in the range of 398.7 ± 28.10 in the Słupsk group (*n* = 17) g and 409.8 ± 27.76 g (*n* = 14) in the Szpęgawa sample. These were not young birds, which can be easily distinguished from adults by the color of their plumage and the appearance of the nostril cere on the beak (Johnston and Janiga 1995). However, the exact age of the captured birds was unknown, as it cannot be determined by the external or internal features of the bird’s body. Sexual maturation of pigeons begins already after they are 3–4 months old (Hetmański 2011). The sex of the pigeons captured for the study was determined according to the type of gonads (presence of testes or ovaries) only after decapitation. There were 7 females and 10 males among the birds captured in Słupsk and 7 females and 7 males in the sample from Szpęgawa. Identification of the sex of pigeons based on the type of gonads is the best method, as sexual dimorphism is poorly visible in this species.

Whole back feathers were plucked from each bird and placed in sealed plastic bags. During sample preparation, sterile scalpels were used and substituted each time to avoid contamination as much as possible. All samples were kept at −20 °C until analysis.

### Tissue isolation

The pigeons were euthanized by decapitation after ether anesthetization and immediately dissected. Approximately 1–3 g of tissues were collected from the heart and skeletal muscles (m. pectoralis) and stored in clean polyethylene vials, which were placed in an ice bath. The tissues were weighed and washed in ice-cold buffer. The minced tissue was rinsed clear of blood with cold isolation buffer and homogenized in a homogenizer H500 (Pol-Eko Aparatura, Poznań, Poland) with a motor-driven pestle on ice. The minced tissue was rinsed to remove blood with cold isolation buffer and homogenized in a homogenizer H500 (POL-EKO, Poland) with a motor-driven pestle on ice. The homogenates were centrifuged at 3000 g for 15 min at 4 °C. The isolation buffer contained 180 mM KCl, 10 mM HEPES, 10 mM EGTA, and 0.5% bovine serum albumin; the pH value was adjusted to 7.3 with KOH. The suspension was then centrifuged for 5 min at 600 g at 0◦C. Finally, the muscle homogenates were kept in the tube on ice until biochemical measurements were performed. The protein concentration in each sample was determined according to Bradford (1976), using bovine serum albumin as a standard. The study was conducted with the consent of the Bioethics Committee (License Number 44/2012).

### Determination of element concentrations

The soil samples were collected at a depth of 1–3 cm. Then, the samples were aggregated and air-dried before storage and analysis. Each soil and feather sample was analyzed in three series. Between different reads, the soil sample was thoroughly mixed within the same bag. The results of three reads were averaged.

The concentrations of chemical elements were analyzed in the feather and soil samples with an X-Ray fluorescence (XRF) analyzer at the Department of Physics, Pomeranian University in Słupsk (Poland). The XRF analyzer, model Sci Sps X-200 from SciAps Inc., was used for determination of the concentrations of chemical elements in the samples. The analyzer is constructed to study elements in different samples such as soil, alloys, precious metals, and some others.

The XRF (X-Ray Fluorescence) analyzer generates an X-ray beam that can be used for irradiating the sample. Interaction of the X ray quanta with the analyzed sample causes characteristic X-ray emission from chemical elements in the sample. The analyses were conducted with an Rh target (50 kV, 600 μA) and polycapillary optics providing a spot size of 25 μm. The X-ray fluorescence signal was collected by two XFlash silicon drift detectors. They provide high spectral resolution of 135 eV measured on the full width at half maximum, FWHM, at 5.95 Mn K-alpha line. The detectors register the spectra of Roentgen fluorescence, or X ray fluorescence, containing information about the presence of chemical elements and their concentrations. Commonly, the K and L series of X-ray fluorescence are used for identification of chemical elements, as they yield the best results. The detectors have an active area of 30 mm^2^ placed at 45° to the X-ray beam. The analyses were carried out under vacuum (20 mbar), using a sampling step of 20 μm and 10 ms dwell time. The apparatus is factory calibrated with 37 standard elements including all measurable pathfinders. The X-ray fluorescence hyperspectral data were processed using PyMca 5.1.3 (Solé et al. 2007) and Datamuncher (Alfeld and Janssens 2015) software. The device software uses either standard methods such as basic parameters for the spectra of the given elements (we used this method in our measurements) or user-generated empirical calibration curves to relate the X-ray spectrum to the element concentrations.

### Reagents and solutions

All reagents used in the current study were purchased from Sigma-Aldrich (Sigma-Aldrich Sp. z o.o., Poznan, Poland) and Avantor Performance Materials Poland S.A. (Gliwice, Poland). The reagents were freshly prepared. All other reagents used were of analytical reagent grade. The Bradford method (1976) with bovine serum albumin as a standard was used for the quantification of proteins. Absorbance was recorded at 595 nm. The enzymatic reactions were started by adding tissue homogenates. The specific assay conditions are presented subsequently. Each sample was analyzed in duplicate.

### Diene conjugates (DK) assay

The diene conjugate level was assessed in the sample with the method proposed by Kamyshnikov (2004). Four mL of a “heptane-isopropanol” mixture were added to 0.2 mL of the homogenate and vortexed vigorously. Next, 1 mL of HCl (pH 2.0) and 2 mL of heptane reagent were added, vortexed, and centrifuged at 3000 rpm for 5 min. The diene conjugate level was read spectrophotometrically at 233 nm and expressed as A_233_ per mL. A mixture with distilled water was used in the blank.

### 2-Thiobarbituric acid reactive substances (TBARS) assay

The level of lipid peroxidation was determined by quantifying the concentration of 2-thiobarbituric acid reacting substances (TBARS) with the Kamyshnikov (2004) method for determination of the malonic dialdehyde (MDA) concentration. This method is based on the reaction of the degradation of the lipid peroxidation product, MDA, with 2-thiobarbituric acid (TBA) at high temperature and acidity to generate a colored adduct measured spectrophotometrically. The nmol of MDA per mg protein was calculated using 1.56·10^5^ mM^−1^ cm^−1^ as the extinction coefficient.

### Total antioxidant capacity assay

The TAC level in the sample was estimated by measuring the TBARS level after Tween-80 oxidation. The level was determined spectrophotometrically at 532 nm by Galaktionova and co-workers (1998). The sample inhibited the Fe^2+^/ascorbate-induced oxidation of Tween-80, resulting in a decrease in the TBARS level. The level of TAC in the sample (%) was calculated for the absorbance of the blank.

### Succinate dehydrogenase (SDH) activity

Succinate dehydrogenase (EC 1.3.99.1) activity was measured spectrophotometrically by following the reduction of potassium ferricyanide (K_3_FeCN_6_) at 420 nm according to the method of Eschenko and Volski (1982) with some modifications. The assay mixture (pH 7.8) contained 0.1 M phosphate buffer, 25 mM EDTA, 0.1 M succinate acid, 150 mM sodium aside, and 25 mM K_3_FeCN_6_. The reaction was initiated by adding 0.5 mL of the homogenate. The enzyme activity was expressed as nmol succinate per min per mg protein.

### Pyruvate dehydrogenase (PDH) activity

Pyruvate dehydrogenase (EC 1.2.4.1) activity was measured spectrophotometrically according to the method proposed by Eschenko and Volski (1982) with some modifications by following the reduction of NAD + to NADH(H^+^) at 340 nm using 140 μM Tris-HCl buffer, pH 7.2, 5 μM potassium pyruvate as a substrate, 5 μM mercaptoethanol as an SH-reagent, 2 μM NAD^+^, and a 0.1% Coenzyme A solution. The reaction was initiated by adding 0.1 mL of the homogenate. The enzyme activity was expressed as nmol per min per mg protein.

### Isocitrate dehydrogenase (ICDH) activity

Mitochondrial isocitrate dehydrogenase (EC 1.1.1.41) activity was measured according to the method developed by Eschenko and Volski (1982) by measuring the reduction of NAD^+^ to NADH(H^+^) at 340 nm. The incubation sample (2.9 mL) contained 100 μM Tris-HCl buffer, pH 7.2, 16 μM sodium isocitrate, 4 μM MnCl_2_, 1 μM NAD^+^, and 2 μM ADP. The reaction was initiated by adding 0.1 mL of the homogenate. The enzyme activity was expressed as nmol per min per mg protein.

### Α-ketoglutarate dehydrogenase (KGDH) activity

Α-ketoglutarate dehydrogenase (EC 1.2.4.2) activity was measured spectrophotometrically according to the method proposed by Eschenko and Volski (1982) by measuring the reduction of 1 μM NAD^+^ to NADH(H^+^) at 340 nm using 300 μM phosphate buffer, pH 7.4 as an assay buffer, 0.45 μM Coenzyme A, 1.5 μM dithiothreitol, 0.2 μM thiamine pyrophosphate, and 6 μM α-ketoglutarate as a substrate. The reaction was initiated by adding 0.1 mL of the homogenate. The enzyme activity was expressed as nmol per min per mg protein.

### Lactate dehydrogenase (LDH) activity

The colorimetric method developed by Sevela and Tovarek (1959) was used for the determination of lactate dehydrogenase (EC 1.1.1.27) activity. Pyruvate is formed through LDH action in the presence of a sample and NAD^+^. The reduction of the NAD^+^ level is coupled with the reduction of l-lactate. Briefly, 0.1 mL of the sample was mixed with 0.3 mL of the NAD^+^ reagent (0.6 mg per sample), 0.8 mL of 30 mM tetrasodium pyrophosphate (pH 8.8), and 0.2 mL of a 450 mM sodium lactate solution (pH 7.5). The samples were incubated at 37 °C for 15 min. The addition of 2,4-dinitrophenyl hydrazine resulted in the formation of the hydrazone complex with ketoacid to form their respective hydrazone derivatives, which were measured colorimetrically at 530 nm. The red color was produced upon the addition of 0.4 M NaOH and was related to the enzyme activity. In the measurement of LDH activity, pyruvate was used as the standard for the calibration graph composition. One unit of LDH was defined as the formation of 1 nmol of pyruvate per min at 370 C incubation per mg of protein.

### Lactate and pyruvate concentrations

Lactate and pyruvate concentrations were measured according to the procedure described by Herasimov and Plaksina (2000). One mL of the sample was added to 6 mL of distilled water and 1 mL of 10% metaphosphoric acid. The mixture was centrifuged at 800 g for 5 min to separate the supernatant. One mL of 25% copper (II) sulfate and 500 mg calcium hydroxide was added to the supernatant, which was then mixed for 30 min. The mixture was centrifuged at 1000 g for 10 min. For the lactate concentration assay, the resulting supernatant was resuspended in 3 mL of p-dimethylamino benzaldehyde (0.5% solved in dimethyl sulfoxide) and 1 mL of 25% NaOH. The mixture was incubated at 37 °C for 45 min, which was then centrifuged at 1000 g for 10 min. The absorbance was measured at 420 nm. A mixture with 0.5% *p*-dimethylaminobenzaldehyde and 25% NaOH was used as a blank. For the pyruvate concentration assay, the resulting supernatant was resuspended in 0.1 mL of 10% copper (II) sulfate, 4 mL of concentrated H_2_SO_4_, and 0.1 mL of 20% hydroquinone dissolved in 96% ethanol, which was then heated in a water bath at 95 °C for 15 min. The absorbance was measured at 430 nm. Calibration curves of lactate (0.1–50 μM) and pyruvate (0.1–50 μM) were used, and the results were expressed in nmol lactate per mg of protein. The lactate-to-pyruvate ratio (L/P) was also calculated.

### ATP level

ATP concentration was measured according to the method developed by Eschenko and Volski (1982). The principle of the method consists in using conjugated systems of two enzymatic reactions. ATP contained in a tissue sample in the presence of hexokinase causes phosphorylation of glucose. The resulting glucose-6-phosphate, in turn, serves as a substrate for the glucose-6-phosphate dehydrogenase reaction. The amount of ATP reaction is equimolar to the amount of NADP^+^ generated in the glucose-6-phosphate dehydrogenase reaction in a pre-prepared deproteinated tissue sample. The assay mixture contained 50 mM triethanolamine buffer, pH 7.5, 7.5 mM NADP, and the enzyme. The amount of ATP produced was determined spectrophotometrically at a wavelength of 340 nm. The ATP content was calculated in µmol per 1 g of tissue.

### Alanine aminotransferase (AlAT) and aspartate aminotransferase (AsAT) activity assay

Alanine aminotransferase (EC 2.6.1.2) and aspartate aminotransferase (EC 2.6.1.1) activities were analyzed spectrophotometrically with a standard enzymatic method described by Reitman and Frankel (1957). The substrates in the reaction were 2 mM α-ketoglutaric acid plus 200 mM l-aspartate (pH 7.4) for AsAT activity and 2 mM α-ketoglutaric acid plus 200 mM l-alanine (pH 7.4) for AlAT activity. The addition of 2,4-dinitrophenyl hydrazine resulted in the formation of the hydrazone complex with the ketoacid to form their respective hydrazone derivatives, which were measured colorimetrically at 530 nm. A red color was produced upon the addition of 0.4 M NaOH. The intensity of the color is related to enzymatic activity. In the measurement of both enzymes, pyruvate is used as the standard for the calibration graph composition. One unit of AsAT or AlAT is defined as the liberation of 1 nmol of pyruvate per min at 37 °C incubation per mg of protein. The De Ritis ratio (AsAT to AlAT activity) was also calculated.

### Lysosomal enzyme assays

#### Tissue isolation for lysosomal enzyme assays

The selected tissues were removed from the pigeons and the tissue samples were excised, weighed, washed in ice-cold buffer, and minced. The minced tissues were rinsed with cold isolation buffer 0.15 M KCl to remove the blood and homogenized in a glass Potter-Elvehjem homogenizer with a motor-driven Teflon pestle on ice. The isolation buffer consisted of 0.25 M sucrose and 2 mM EDTA; the pH value was adjusted to 7.0 with KOH. The homogenates of several muscles 20% (w/v) were prepared for the next differential centrifugation according to the method described by DeMartino and Goldberg (1978). After centrifugation, the supernatant fractions were saved and used after resuspension in 50 mM acetic acid/sodium acetate buffer, pH 5.0. The isolated fractions were homogenized and subjected to two freeze-thaw cycles.

#### Lysosomal enzyme assays

The activity of lysosomal alanyl aminopeptidase (EC 3.4.11.2) and leucyl aminopeptidase (EC 3.4.11.1) was determined spectrophotometrically according to McDonald and Barrett (1986) as Fast Blue BB salt (4-benzoyloamino-2,5-diethoxybenzene-diazonium chloride) derivatives at 540 nm. The reaction was initiated by adding 500 μL of substrate incubation media with DMF to 50 μL of the sample (Serva, Germany), 60 min incubation at 37 °C, pH 6.0. Next, 500 μL of stop buffer consisting of Fast Blue BB salt dissolved in 2% Tween 20 (Sigma, USA) were added. Absorbance was measured at 540 nm. l-alanyl-2-naphtylamine in 0.1 M PBS buffer was used as a substrate for determination of alanyl aminopeptidase activity and l-leucyl-2-naphtylamine in 0.1 M PBS pH 7.0 buffer was used as a substrate for determination of leucyl aminopeptidase activity. The activities of other lysosomal enzymes such as acid phosphatase (EC 3.1.3.2.) and β-N-acetylglucosaminidase (EC 3.2.1.30.) were determined spectrophotometrically as 4-nitrophenyl derivatives at 420 nm with the method used by Barrett and Heath (1977). The activities of enzymes were expressed in nmol per h per mg protein.

### Statistical analysis

The basic statistical analyses (significance of regression slopes, analysis of variance for significance between cities and between tissues for metals and enzyme activity, distributing testing) were carried out using the Statistica 13.3 package (StatSoft, Krakow, Poland). The data were tested for homogeneity of variance using Levene’s test of equality of error variances. Normality was checked by the Kolmogorov-Smirnov test.

The results are expressed as mean ± S.D. Significant differences among the means were measured using a multiple range test at min. *P* < 0.05. Not normally distributed data were log-transformed. Student t-tests with 95% confidence intervals (α = 0.05) were applied to determine the significance of differences in the element concentrations between the types of regions and the significance of differences in element levels in soil and feathers of birds as well as enzyme activities in the tissues (skeletal muscles and cardiac tissue) of birds from the different regions.

The correlation of parametric values was based on Pearson’s regressions analysis using the multiple regression module. The correlation and regression analysis comprised the correlation coefficient (r), regression equation, and significance of these dependencies (P). The arithmetic means of the element concentrations and enzyme activities in skeletal muscle and cardiac tissues were estimated using two-way ANOVA. The use of multivariate significance tests of the main effects (type of the environment, type of tissues, and their combined effects) helped to determine statistically significant relationships for all three values. In the model approach, to combine the impact of two factors (environment and tissues), we adopted a two-way classification model, estimating the value of the dependent variable, the mean, the main effect of the environmental factor, main effect of the tissue factor, the effect of the interaction of the environment and tissue type factors, and the experimental random error.

We used the coefficients of multiple correlation analysis (R), the coefficient of determination (R^2^), and its corrected form reduced by random errors (R^2^ adjusted) in the data analysis for the description of the full model. The SS test was used to describe the share of all analyzed biomarkers of oxidative stress and biochemical parameters for assessment of the antioxidant defenses with the F test and its significance (Zar 1999).

## Results

### Metals

The mean bodyweight of the pigeons was 398.7 ± 28.10 in the Słupsk group (*n* = 17) g and 409.8 ± 27.76 g (*n* = 14) in the Szpęgawa sample. The pigeons from Szpęgawa were slightly heavier than those caught in Słupsk, but the difference was not statistically significant (*p* = 0.281).

The first stage of the investigations consisted in determination of the mean concentration of elements in twelve soil samples collected from four sites at the perimeters of two locations with different pollution gradients (non-polluted – Słupsk, polluted – Szpęgawa, Pomeranian region, northern Poland). The Student t-test was used to compare the results and determine statistically significant correlations between the element levels in the soil from these locations. The concentration (g/kg) of Al was higher by 109% (*F* = 4.709, *p* = 0.000), Ti – by 21% (*F* = 1.941, *p* = 0.044), Mn – by 219.5% (*F* = 1.417, *p* = 0.000), Fe – by 12.8% (*F* = 1.782, *p* = 0.009), Pb – by 482.6% (*F* = 9.941, *p* = 0.000) in the polluted area, compared to the unpolluted site. It should be noted that the Si concentration was more than twofold higher (*F* = 4.762, *p* = 0.029) in the soil samples from the unpolluted area and this data was significant (Table 1).

**Table 1:** Mean concentrations of elements (g/kg) ± standard deviation (S.D.) in soil samples collected from the different areas (Polluted area – Szpęgawa, Non-polluted area – Słupsk, Pomeranian region, Northern Poland)

Elements	Polluted areas (*n* = 12)	Non-polluted areas (*n* = 12)	*p*	*F*
Al	80.28 ± 28.11	38.43 ± 12.95	0.000	4.709
Si	113.89 ± 156.89	230.41 ± 71.89	0.029	4.762
Ti	34.80 ± 7.97	28.76 ± 5.72	0.044	1.941
Mn	12.30 ± 2.08	3.85 ± 1.75	0.000	1.417
Fe	708.06 ± 57.64	627.88 ± 76.95	0.009	1.782
Ni	4.74 ± 1.01	4.56 ± 0.93	0.647	1.169
Cu	3.89 ± 0.59	4.18 ± 1.12	0.427	3.607
Zn	9.44 ± 1.64	24.25 ± 9.02	0.000	30.123
Zr	26.06 ± 7.69	46.66 ± 19.0	0.002	6.100
Pb	28.49 ± 9.11	4.89 ± 2.89	0.000	9.941

Data collected and analyzed from 12 independent samples. Student t-tests with 95% confidence intervals (*p* = 0.05) were applied to determine the significance of differences between element concentrations in the different areas and the significance of differences in element levels in the soil

*p*, significant site-dependent differences in element levels; *F*, variance level

The mean concentration of elements (g/kg) in pigeon feathers collected from sites along the heavy metal pollution gradient exhibited similar tendencies in the element concentration levels, as indicated by the Student *t*-tests: the Si and Pb levels were higher by 4.1% (*F* = 5.024, *p* = 0.001) and by 319.4% (*F* = 2.545, *p* = 0.001), respectively, in the polluted area than in the unpolluted locality. Simultaneously, in feathers of pigeons from the unpolluted area, a statistically significant increase in the level of Fe (by 78.8%, *p* = 0.007) and Cu (by 23.3%, *p* = 0.047) was observed (Table 2). The analysis of the data obtained allowed us to compare element concentrations in the birds’ habitat with those in the feathers and to suggest primarily lead-induced intoxication of pigeons.

**Table 2:** Mean concentrations of elements (g/kg) ± standard deviation (S.D.) in feathers of pigeons sampled from the different areas (Polluted area – Szpęgawa, Non-polluted area – Słupsk, Pomeranian region, Northern Poland)

Elements	Polluted areas	Non-polluted areas	*p*	*F*
Al	51.83 ± 9.69	48.58 ± 13.29	0.452	1.884
Si	896.18 ± 15.42	860.72 ± 34.56	0.001	5.024
Fe	11.82 ± 7.25	21.13 ± 10.11	0.007	1.941
Ni	2.13 ± 0.60	2.54 ± 0.66	0.080	1.211
Cu	4.60 ± 1.36	5.67 ± 1.48	0.047	1.184
Zn	33.77 ± 10.77	61.64 ± 16.37	0.000	2.308
Pb	5.62 ± 3.92	1.34 ± 2.46	0.001	2.545

Student *t*-tests with 95% confidence intervals (*p* = 0.05) and *F*-tests of equality of variances were applied to determine the significance of differences between element concentrations depending on the study area

### Pro/antioxidant balance

Free-radical oxidation is a complex, multi-stage physiological process that regulates the cellular activity and body functions and has several interrelated mechanisms (Halliwell and Gutteridge 2007; Liochev 2013). Lipid peroxidation is one of these processes, which is enhanced when the antioxidant activity of a cell decreases due to an increase in the content of heavy metals (Ercal et al. 2001). Therefore, we have consistently analyzed the initial and final stages of this process as well as the total antioxidant capacity of the pigeon muscle tissues. The relevant data are shown in Table 3.

**Table 3:** Levels of diene conjugates (DK, nmol mg^−1^ protein), TBARS (nmol MDA·mg^−1^ protein), and total antioxidant capacity (TAC, %) in the skeletal muscle (SM) and cardiac tissues (CT) of pigeons nesting in polluted (P, Szpęgawa) and non-polluted areas (NP, Słupsk) of the Pomeranian region, northern Poland

Biomarkers	Parameters					
	Areas	Tissues	Mean ± S.D.	Minimum	Maximum	Skewness
DK, nmol mg^−1^ protein	P	SMT	64.35 ± 12.48*	47.19	91.67	−0.18
	P	CT	76.13 ± 8.74**	63.81	102.22	0.64
	NP	SMT	34.35 ± 12.48	17.19	51.67	−0.22
	NP	CT	44.09 ± 9.10	29.58	69.64	0.89
TBARS, nmol·mg^−1^ protein	P	SMT	59.33 ± 11.22	38.97	78.75	−0.18
	P	CT	57.96 ± 27.46**	23.33	112.56	0.64
	NP	SMT	47.57 ± 11.58	22.23	66.11	−0.22
	NP	CT	21.95 ± 8.66	13.53	37.46	0.89
TAC, %	P	SMT	25.66 ± 9.55*	11.58	50.41	1.01
	P	CT	32.82 ± 13.53	15.70	57.85	0.45
	NP	SMT	44.93 ± 18.62*	20.66	79.01	0.27
	NP	CT	50.01 ± 9.10	25.75	62.02	−1.04

Student *t*-tests with 95% confidence intervals (*α* = 0.05) were applied to determine the significance of differences in the level of oxidative stress biomarkers between the types of areas

*P < 0.05

**P < 0.01

***P < 0.001

The analysis revealed statistically significant differences in the lipid peroxidation biomarkers at the initial (content of diene conjugates, DK) and final stages (TBARS content) in the two types of muscle tissues (skeletal muscles and cardiac tissue) differing in their structural and morphofunctional characteristics. Higher DK and TBARS levels were observed in the birds from the polluted area (Szpęgawa) compared to the non-polluted habitat (Słupsk). It should be noted that the stable tendency toward a pollution-induced increase in lipid peroxidation in cardiac and skeletal muscle tissues was accompanied by reduced total antioxidant capacity of these tissues in the studied birds.

### Enzymes and substrates of energetic metabolism

**Fig. 2:**
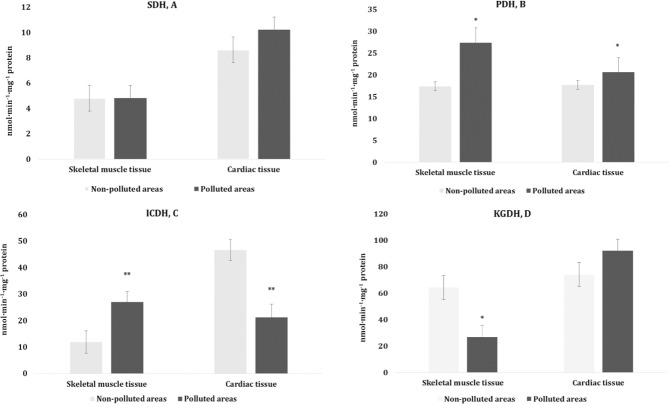
Activities of succinate dehydrogenase (SDH, A, nmol·min^−1^·mg^−1^ protein), pyruvate dehydrogenase (PDH, B, nmol·min^−1^·mg^−1^ protein), isocitrate dehydrogenase (ICDH, C, nmol·min^−1^·mg^−1^ protein), and α-ketoglutarate dehydrogenase (KGDH, D, nmol·min^−1^·mg^−1^ protein) in the muscle tissues (skeletal muscle and cardiac tissues) of pigeons nesting in polluted (Szpęgawa) and non-polluted areas (Słupsk) of the Pomeranian region, northern Poland. Student t-tests with 95% confidence intervals (*α* = 0.05) were applied to determine the significance of differences in the level of oxidative stress biomarkers between the types of areas. *P < 0.05

Krebs cycle enzymes are a source of reducing equivalents that supply the respiratory chain of the mitochondria and form the electrochemical potential gradient as a universal form of energy (Hoppeler and Fluck 2003; Sheeran and Pepe 2017). This approach to assessment of energy metabolism is one of the most important indicators of the functional activity of body tissues (heart, muscles) in animals from a contaminated environment. We chose the four main enzymes of the Krebs cycle [succinate dehydrogenase (SDH), isocitrate dehydrogenase (ICDH), pyruvate dehydrogenase (PDH), and α-ketoglutarate dehydrogenase (KGDH)] to analyze this issue. The activities of enzymes associated with the Krebs cycle exhibited changes presented in Fig. 2. The highest PDH activity was observed in the skeletal muscle and cardiac tissues of pigeons from the polluted area compared to those from the non-polluted habitat (Fig. 2B). We also observed the highest ICDH activity in the skeletal muscles and the lowest activity in the cardiac tissue of birds from the polluted area (Fig. 2C). Statistically significant changes in the KGDH activity in the muscles were observed (Fig. 2D). Thus, Thus, multidirectional functions and different metabolic pathways of the analyzed Krebs cycle enzymes were shown in the present study.

**Fig. 3:**
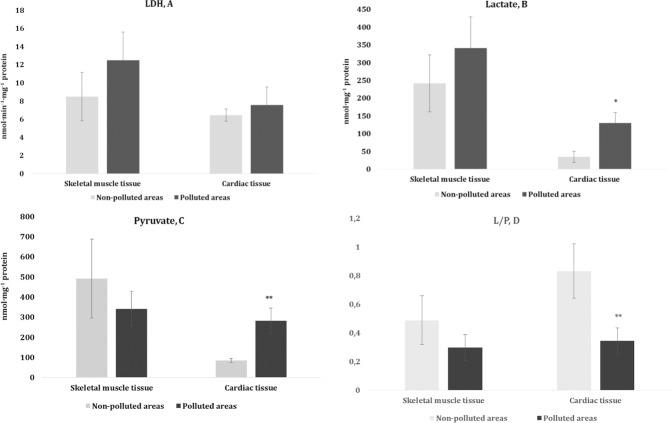
Lactate dehydrogenase (LDH, A, nmol·min^−1^·mg^−1^ protein) activity, levels of lactate (B, nmol·mg^−1^ protein) and pyruvate (C, nmol·mg^−1^ protein), and the lactate/pyruvate ratio (L/P, D) in the muscle tissues (skeletal muscle and cardiac tissues) of pigeons nesting in polluted (Szpęgawa) and non-polluted areas (Słupsk) of the Pomeranian region, northern Poland. Student t-tests with 95% confidence intervals (α = 0.05) were applied to determine the significance of differences in the level of oxidative stress biomarkers between the types of areas. *P < 0.05; **P < 0.01; ***P < 0.001

**Fig. 4:**
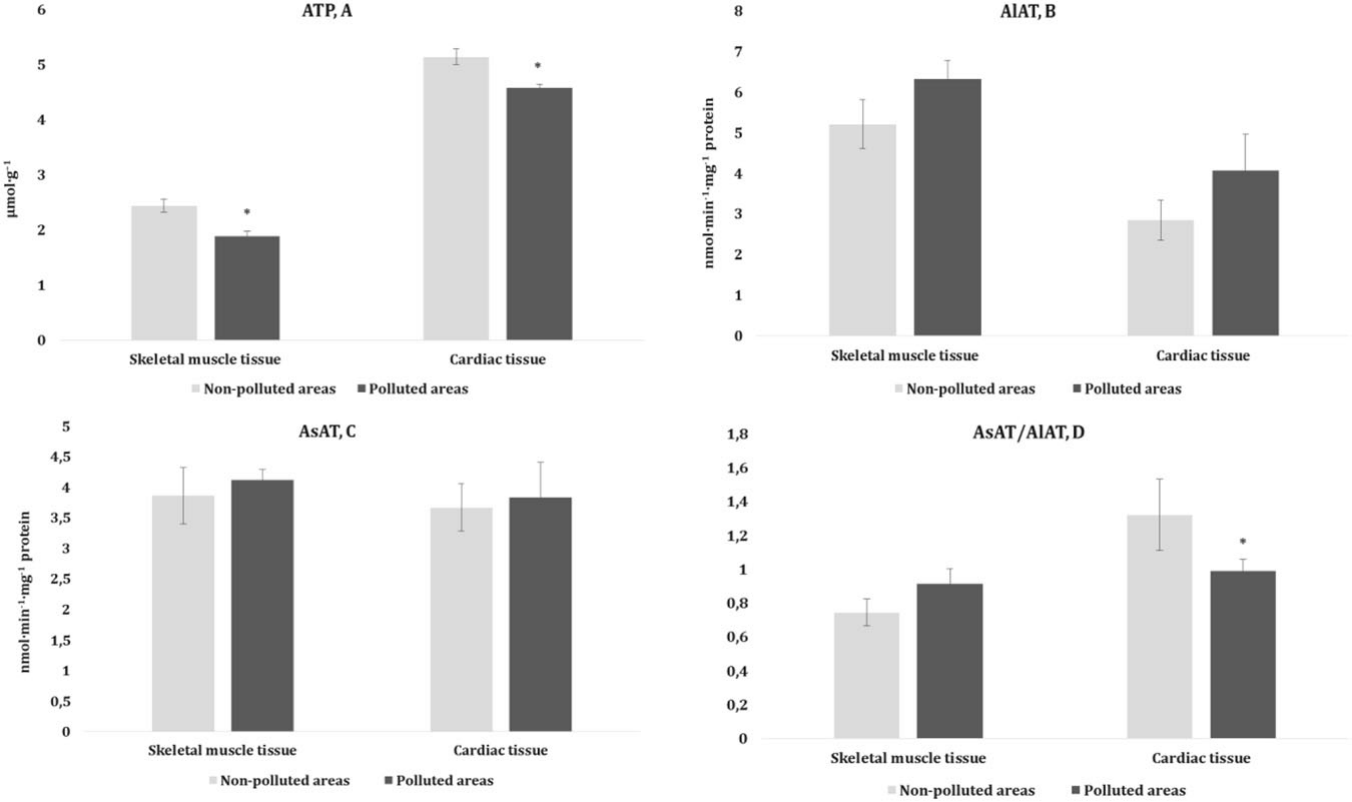
ATP concentration (A, μmol·g^–1^), activities of AlAT (B, nmol·min^−1^·mg^−1^ protein) and AsAT (C, nmol·min^−1^·mg^−1^ protein), and the AsAT/AlAT ratio in the (D) muscle tissues (skeletal muscle and cardiac tissues) of pigeons nesting in polluted (Szpęgawa) and non-polluted areas (Słupsk) of the Pomeranian region, northern Poland. Student t-tests with 95% confidence intervals (*α* = 0.05) were applied to determine the significance of differences in the level of oxidative stress biomarkers between the types of areas. *P < 0.05

Another focus of our study was the determination of the level of lactic acid and its associated metabolites such as pyruvate. These data are presented in Fig. 3. Our hypothesis was that lactic acid plays the role of a metabolic plug in animal tissues with an intensive type of metabolism, especially in birds. It is known that the main route of lactate metabolism is the easily reversible lactate dehydrogenase reaction (Elustondo et al. 2013). Another point of view is that lactate can be seen as a tissue reserve of actively metabolizing pyruvate (Hochachka and Somero 2002). The reversibility of the lactate dehydrogenase reaction and the high activity of the enzyme allows lactate and pyruvate substrates to play an important role in controlling the ratio of oxidized and reduced NAD forms. In the current study, the lactate and pyruvate levels associated with glycolysis and the Krebs cycle exhibited changes only in the cardiac tissue of the pigeons from the polluted area. Thus, a statistically significantly decrease in the lactate/pyruvate ratio was observed for the cardiac tissue of the birds from the polluted area. We analyzed the ATP level in tissues with different functional and metabolic properties in the organism of birds. The relevant data are shown in Fig. 4. The quantitative determination of the ATP level in the tissues revealed a statistically significant decrease in the ATP level in both types of tissue in birds nesting in the polluted area.

Another important aspect of this part of the study was the determination of alanine and aspartate aminotransferase activity in the skeletal muscle and cardiac tissues of the pigeons. The transaminase reactions between keto-acids of the Krebs cycle (α-ketoglutarate and oxaloacetate) and amino acids play an important role in the linking of metabolic pathways of amino acids, lipids, and carbohydrates. Equally important is the fact that we showed changes in KGDH and a relationship between this enzyme, due to its substrate specificity (*α*-ketoglutarate as the reaction substrate), and two other enzymes, i.e., alanine and aspartate aminotransferases. Therefore, to acquire a holistic understanding of changes in the energy profile, the next step of our study was to determine the activity of both amino acid transferases and their ratio (de Ritis ratio), which have diagnostic importance. In the current study, no statistically significant changes in the AlAT and AsAT activities were noted, but the AsAT/AlAT ratio was decreased in the cardiac tissue of pigeons from the polluted area.

### Lysosomal enzymes

**Fig. 5:**
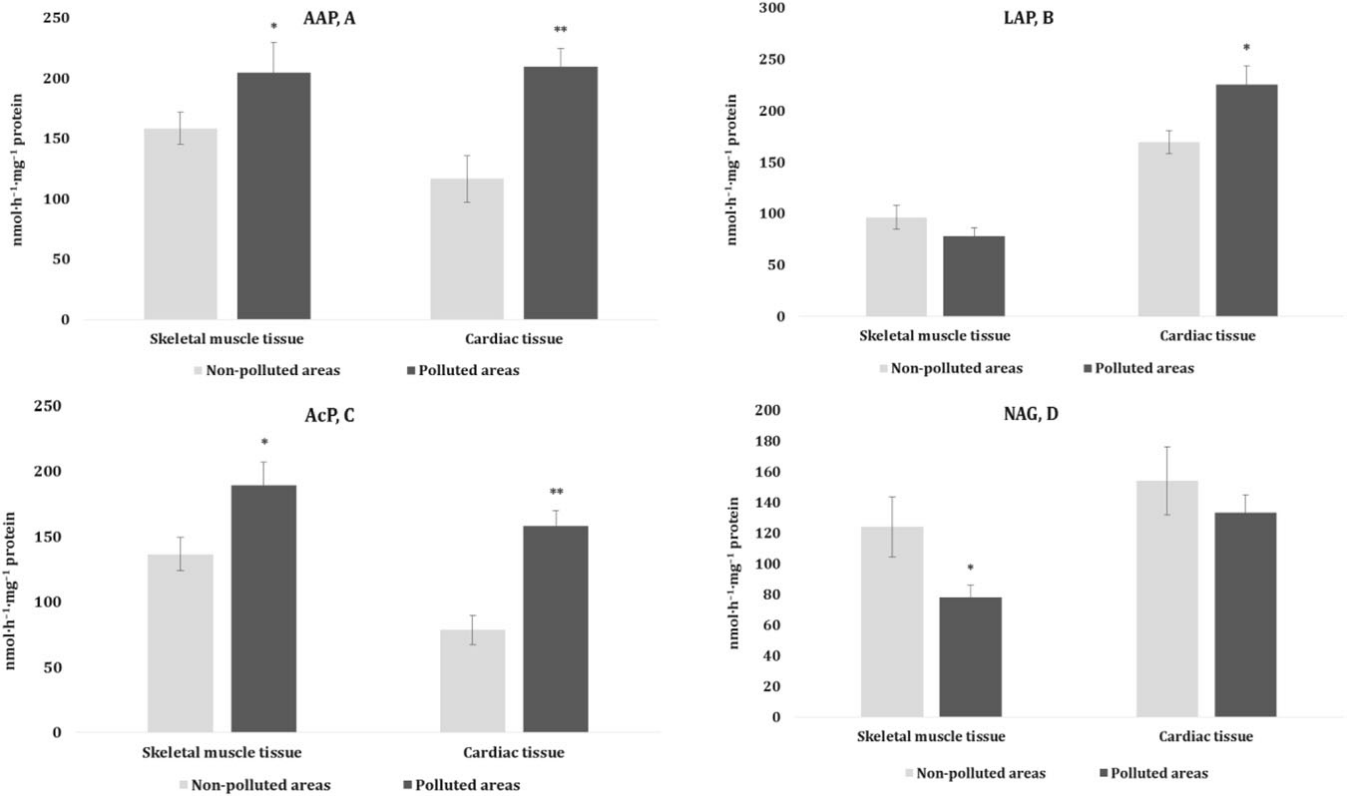
Activities of lysosomal alanyl aminopeptidase (AAP, A, nmol·h^–1^·mg^–1^ protein), leucyl aminopeptidase (LAP, B, nmol·h^–1^·mg^–1^ protein), acid phosphatase (AcP, C, nmol·h^–1^·mg^–1^ protein), and β-N-acetylglucosaminidase (NAG, D, nmol·h^–1^·mg^–1^ protein) in the muscle tissues (skeletal muscle and cardiac tissues) of pigeons nesting in polluted (Szpęgawa) and non-polluted areas (Słupsk) of the Pomeranian region, northern Poland. Student t-tests with 95% confidence intervals (*α* = 0.05) were applied to determine the significance of differences in the level of oxidative stress biomarkers between the types of areas. *P < 0.05; **P < 0.01

The next stage of our study was to determine changes in the lysosomal apparatus in the skeletal muscles and cardiac tissue of pigeons induced by proteolytic enzymes and to evaluate tissue specificity. The results are shown in Fig. 5. The mechanisms affecting the activity of proteolytic enzymes and their high lysosomal activity can be triggered by intensification of lipid peroxidation. The analysis of the alanyl aminopeptidase (AAP) activity, i.e., a membrane-bound enzyme, showed that it was altered significantly in the skeletal muscles and cardiac tissue in birds from the polluted area compared to those from the non-polluted one. Similar tendencies were observed in both types of tissue for the acid phosphatase (AcP) and leucyl aminopeptidase (LAP) activity in the cardiac tissue. The β-N-acetylglucosaminidase (NAG) activity in the muscle tissue was decreased.

Multivariate tests of significant main effects (environments and tissues) with the sigma-restricted parameterization of the effective hypothesis decomposition by two-way ANOVA were used for analysis of the biochemical markers of the oxygen-dependent pathways in the pigeon muscle tissues. Our results indicated significant dependencies of such main factors as the environment (Wilks test value = 0.178, *F* = 17.406, *p* = 000) and the tissue type (Wilks test value = 0.049, *F* = 13.23, *p* = 0.000) and their combination (Wilks test value = 0.084, *F* = 34.56, *p* = 0.000).

We used the SS test in two-way ANOVA for the whole model vs. residual SS of the parameters for a full model data for two types of tissue (cardiac and skeletal muscle tissues) and two types of environment for the oxidative stress biomarkers, levels of substrates, and activities of metabolic and lysosomal enzymes (Table 4). For analysis of these three independent processes in the holistic model, the multiple correlation coefficient (R), the coefficient of determination (R^2^), and adjusted R^2^ were used. These tests allowed us to formulate the following conclusions on the role of each parameter analyzed in an integral model. These dependencies were as follows: TBARS > TAC > DK, designating TBARS as an effective biomarker of oxidative stress. The dependencies of the described enzymes and metabolites of the oxygen-dependent pathways were as follows: AlAT > Pyruvate > ICDH > AsAT/AlAT ratio > Lactate > ATP > PDH > L/P ratio > SDH > KGDH > LDH > AsAT, indicating the key role ICDH and AsAT in assessment of the predominance of aerobic or anaerobic metabolic pathways in pigeon muscle tissues. The role of the lysosomal enzymes shown by the SS test allowed us to propose the following conclusions on the dependencies: AAP > AcP > LAP > NAG, indicating AAP and AcP as effective biomarkers of structural and functional lysosomal processes.

**Table 4:** SS test of the whole model vs. residual SS of parameters for a full model data for two types of tissue (skeletal muscle and cardiac tissues), two types of areas for oxidative stress biomarkers, activities of metabolic and lysosomal enzymes, and level of substrates

Parameters	Multiple R	Multiple R^2^	Multiple adjusted R^2^	*F*	*P*
DK	0.417	0.172	0.130	4.221	0.009
TBARS	0.661	0.437	0.409	15.80	0.000
TAC	0.589	0.347	0.315	10.83	0.000
SDH	0.550	0.303	0.269	8.86	0.000
PDH	0.578	0.335	0.302	10.24	0.000
ICDH	0.768	0.591	0.571	29.41	0.000
KGDH	0.458	0.210	0.171	5.427	0.002
LDH	0.418	0.175	0.134	4.321	0.007
Lactate	0.663	0.439	0.412	15.95	0.000
Pyruvate	0.776	0.602	0.582	30.81	0.000
ATP	0.627	0.393	0.332	6.422	0.000
L/P	0.557	0.310	0.276	9.170	0.000
ALT	0.804	0.646	0.629	37.22	0.000
AST	0.156	0.024	0.023	0.508	0.678
AST/ALT	0.751	0.564	0.543	26.36	0.000
AAP	0.874	0.764	0.740	32.11	0.000
LAP	0.824	0.679	0.647	21.00	0.000
AcP	0.856	0.732	0.705	27.18	0.000
NAG	0.381	0.145	0.058	1.682	0.086

In the current study, we observed statistically significant dependencies between the values of enzyme activities and the levels of metabolites of the oxygen-dependent pathway and between the lysosomal enzyme activity and the level of lipid peroxidation in muscle tissues using correlation and regression analysis (Table 5). These correlations varied according to the types of tissue (skeletal muscle and cardiac tissues) and types of the environment (polluted, non-polluted).

**Table 5:** Correlative dependencies of parameters for two types of tissue (skeletal muscle and cardiac tissues), two types of areas for oxidative stress biomarkers, activities of metabolic and lysosomal enzymes, and level of substrates

Tissues	Non-polluted area			Polluted area		
	Dependencies	R	*P*	Dependencies	R	*P*
**Skeletal muscle tissue**	Al – ICDH	0.551	0.022	Al – ICDH	0.551	0.022
	Fe – Lactate	–0.514	0.035	Pb – AsAT/AlAT	–0.542	0.035
	Fe – Pyruvate	–0.497	0.042	Pb – LDH	0.630	0.007
	Pb – AsAT/AlAT	–0.5158	0.033	AsAT/AlAT – ICDH	0.551	0.022
	Pb – LDH	0.641	0.006	LDH – PDH	0.607	0.010
	TAC – Si	0.495	0.044	KGDH – SDH	0.538	0.022
	TAC – Fe	–0.561	0.019	AAP – TBARS	0.576	0.012
	TAC – Pyruvate	0.573	0.016	Pb – AcP	0.622	0.005
	LDH – PDH	0.607	0.010	Al – Si	−0.833	0.000
	KGDH-SDH	–0.538	0.026	Al – Zn	0.618	0.008
	AcP – TAC	–0.641	0.006	Cu – Si	−0.698	0.002
	Pb – LDH	0.630	0.007	Cu – Fe	0.618	0.008
**Cardiac tissue**	Fe – AsAT	0.501	0.041	Ni – Si	–0.824	0.000
	Pb –AlAT	0.513	0.035	Ni – TAC	–0.594	0.025
	TBARS – ICDH	0.659	0.004	Si – Cu	–0.632	0.015
	TAC – Lactate	0.601	0.011	Si – Zn	–0.671	0.009
	TAC – PDH	0.540	0.025	Pb – SDH	–0.549	0.022
	ICDH – Pyruvate	–0.503	0.040	TBARS – TAC	0.578	0.030
	L/P – LDH	–0.497	0.042	Zn – Ni	0.678	0.008
	LAP – TAC	–0.538	0.016	TBARS – AcP	0.632	0.005
	Al – Ni	0.631	0.007			
	Zn – Si	–0.896	0.000			

The significance of differences in the different areas and tissues studied was examined using ANOVA for correlation test

## Discussion

The objective of the current study was to determine the relationships between key enzymes and substrates involved in oxygen-dependent processes and lysosomal functioning in the cardiac and skeletal muscle tissues of pigeons from areas with different levels of contamination. Our results confirmed that Pb from anthropogenic sources, including traffic, industrial emissions, and coal sources mainly contributed to these processes (Frantz et al. 2012; Lodenius and Solonen 2013; Cui et al. 2016). A prospective focus of such research is to elucidate the mechanisms of regulation of the activity of oxygen-dependent and lysosome proteolytic processes. These studies determine the importance of proteolysis stimulation in the genesis of oxidative stress caused by heavy metals and other pollutants in birds (Pizzino et al. 2017).

There are several novel findings in our study. First, the results show that the concentration of Pb is higher in feathers of pigeons living in polluted environments. This is probably related to the higher Pb contamination of soils in polluted regions caused by the high traffic on the A1 motorway and coal sources (Hoff Brait and Antoniosi Filho 2011). This phenomenon is observed in the Pomeranian regions of northern Poland studied in this paper, where the level of Pb is relatively high. Pb is accumulated in the blood of chicks during growth, mostly in bones and feathers, and its toxic effects on their organism are intensified with age (Williams et al. 2018).

Pb and other toxic metals primarily disturb growth, reduce the hemoglobin content, displace biologically necessary elements, and have an antagonistic effect on the metabolism of these elements (Cui et al. 2016; Stauffer et al. 2017). The important role of Pb in these processes was also confirmed by the results of our statistical analysis in the correlations between oxidative stress biomarkers, energetic metabolites, and enzymes in the non-polluted environment: Pb – AsAT/AlAT and Pb – LDH in muscle tissue and Pb – AlAT in cardiac tissue. The other metals in the polluted area exhibited correlations with metabolism parameters in the skeletal muscle tissue, i.e. Al – ICDH, Pb – AsAT/AlAT, and Pb – LDH, while the following correlations were observed in the cardiac tissue: Pb – SDH and Ni – TAC. The analysis of tissue of birds from the contaminated environment revealed statistically significant correlations between the level of lead and the biomarkers of energy metabolism: Pb – AsAT/AlAT, Pb – LDH, Pb – AcP, and Pb – SDH (Table 5).

Secondly, our results revealed changes in the activity of metabolic enzymes caused by the modification of energy metabolism pathways, especially in the case of birds nesting in the polluted area. Metals exert toxic effects if they enter into biochemical reactions in which they are not normally involved (Rehman et al. 2018; Kanwal et al. 2020). Thus, redistribution of energy substrates (decreased level of ATP production in cardiac and skeletal muscle tissues) and metabolites (estimated by the level of lactate and pyruvate, as well as the main enzymes of the Krebs cycle) resulted in changes in the levels of substrates for aerobic and anaerobic metabolic pathways and, accordingly, intensification of lipid peroxidation. The analysis showed TAC – Si and TAC – Fe correlations in the skeletal muscle tissue and LAP – TAC, Fe – AsAT, TBARS – ICDH, TAC – Lactate, and TAC – PDH correlations in the cardiac tissue of pigeons from the non-polluted environment (Table 5).

Elucidation of the relationships between metals and metabolic factors can be useful for targeted mitigation of damage to the organism. Based on the course of states associated with increased activity of proteolytic processes, it is possible to assess the dynamic balance of proteinase/inhibitors in the organism. Recent research has shown that a change in this balance promotes development of many kinds of pathology, increases stress disorder, and has systemic consequences for ecosystems and humans. The majority of researchers have found that an increase in the activity of lysosomal enzymes, especially proteolytic enzymes, under stress in samples, is involved not only in the destruction of proteins, including matrix proteins, but also in limited proteolysis (Kaur and Debnath 2015; Koh et al. 2019).

Thirdly, the interaction between chemical elements and antioxidant activity plays an important role in the physiological response of synanthropic birds in their environment (Koivula and Eeva 2010). However, various heavy metals and disturbed transfer of macroelements in the environment have their differential ecophysiological impact upon the level of pro- and antioxidant activity of enzymes, development of lipid peroxidation, and induction of lysosome destructive processes (Cui et al. 2013).

Fourthly, our data regarding the lipid peroxidation processes in the muscle tissue of pigeons nesting in areas with different levels of contamination analyzed by the two-way ANOVA tests confirmed the influence of the types of tissue and birds’ habitat and the combination of these factors. Such dependencies regarding the activity of alanine and aspartate transaminases, especially in the cardiac tissue, lactate dehydrogenase, and succinate dehydrogenase, as well as metabolites involved in energy-related cellular metabolic changes (e.g., lactate and pyruvate) (Zoremba et al. 2014), were observed.

The results of multiple correlation analyses revealed that the biochemical markers of oxygen-dependent pathways in the muscle tissue are dependent on the level of lysosome functioning. We assumed that the value of the proteolytic activity in the tissues of both groups (non-polluted and polluted) changed in direct proportion to the increase in oxidative stress, which in turn was induced by the level of contamination and the pollutant accumulation in the pigeon feathers. We can also suggest that anthropogenic processes and activities (car traffic, using coal sources in homes) may play an important role in bioaccumulation and transfer of chemical elements in various types of environments, but this is not associated with the type of region. There were AcP – TAC and LAP – TAC correlations in the skeletal muscle and cardiac tissue of pigeons from the non-polluted environment. In turn, Pb – AcP, TBARS – AAP, and TBARS – AcP correlations were found in the tissues of birds nesting in the polluted area (Table 5).

The disturbances caused by the exposure to heavy metals, namely lead, and the disturbances in the activities of enzymes related to energy metabolism in the skeletal muscle and cardiac tissues of pigeons from the polluted area may be associated with increased oxidative stress, reduced energy sources, plasticity of metabolic reserves, alterations in energy apparatus, and disturbance in carbohydrate metabolism, as demonstrated in the current study. Previous studies have shown that monovalent metals are almost completely filtered in glomeruli and actively reabsorbed in renal tubules, competing with K^+^ and Na^+^ ions. Bivalent metals (As, Pb, Hg, and Cr) bind to the sulfhydryl groups of specific and non-specific proteins that have a transport function (Pratush et al. 2018). Metal-induced oxidative stress also decreases the stability of the lysosomal membranes, leading to cell death, as shown in several studies (Britton 1996; Baird et al. 2006; Kurz et al. 2007).

Transport proteins can increase the nephrotoxicity of metals when they are excessively supplied to the body (Goering 1993; Avila-Rojas et al. 2019). Lead is an osteotrophic element replacing calcium in hydroxyapatite crystals. When supplied with food and water, lead is differently distributed in the body depending on the muscle type. When it enters the body via the oral pathway, lead is absorbed in the intestine and reaches the liver, from where it enters the duodenum again with bile. One part of lead is reabsorbed and the other part is removed with feces. If it enters the respiratory tract, lead quickly reaches the blood flow and its effect is maximized. Lead is excreted from the blood by the kidneys but some is deposited in the bones. Lead inhibits the action of many enzymes and the incorporation of iron into the body, resulting in a dramatic increase in the free protoporphyrin level in the urine. The toxic effect of lead is largely related to its ability to form complexes with ligands containing sulfhydryl and carboxylic groups in proteins, imidazole derivatives, and phosphate ions (Kim et al. 2014; Williams et al. 2018).

Metals can be deposited in the lysosome matrix as a result of the process of complexation with anion groups and can compete with calcium and magnesium ions for binding to the active centers of the proton pump (Dell’Antone 1988). Heavy metal ions entering the cell in excessive amounts lead to various structural and functional disorders (Rehman et al. 2018). The main causes of these changes include inactivation of enzymes due to the formation of complexes of heavy metal ions with groups of proteins, induction of hydroxyl radicals and superoxide anion, activation of lipid peroxidation, and lysosomal dysfunction (Koh et al. 2019).

The mitochondrial enzyme activity disruptions identified in the present study may cause alterations in the energy metabolism in muscle tissues. When comparing the lead-induced metabolic disturbances in the activity of the main enzymes of the Krebs cycle and the high levels of oxidative stress, more severe damage to the cardiac tissue compared to the muscles was observed. The experiment revealed a decrease in ATP production in both types of muscle tissue. Since the processes of substance transport, secretion, and biosynthesis as well as inactivation of xenobiotics and oxidative stress are energy-dependent, it can be assumed that ATP deficiency leads to disruptions in metabolic processes. The latter phenomenon is undoubtedly related to a decrease in the physiological welfare of birds and induction of pathological processes.

## Conclusions

Our studies have shown that the presence of lead in the birds’ habitat areas activated lipid peroxidation, destabilized the lysosomal membranes, and, as a result, increased the activity, functioning intensity, and activation of mitochondrial enzymes of the Krebs cycle resulting in energy deficiency (reduction of ATP levels) in cardiac and skeletal muscle tissues. Our results indicate an increased level of oxidative stress in the organisms of pigeons living in polluted areas. The increased level of lipid peroxidation modified total antioxidant capacity and altered the function of enzymes of the Krebs cycle and associated metabolic pathways such as lactate and pyruvate as well as aminotransferase activities in the muscles of pigeons nesting in polluted areas.
